# Draft Genome Sequence of *Flavobacterium* sp. Strain PL002, Isolated from Antarctic *Porphyra* Algae

**DOI:** 10.1128/MRA.00063-21

**Published:** 2021-02-25

**Authors:** Chui Peng Teoh, Paris Lavin, Nazalan Najimudin, Ping Chin Lee, Lavinia Iancu, Cristina Purcarea, Clemente Michael Vui Ling Wong

**Affiliations:** a Biotechnology Research Institute, Universiti Malaysia Sabah, Kota Kinabalu, Sabah, Malaysia; b Departamento de Biotecnología, Facultad de Ciencias del Mar y Recursos Biológicos, Universidad de Antofagasta, Antofagasta, Chile; c Laboratorio de Complejidad Microbiana y Ecología Funcional, Instituto Antofagasta, Universidad de Antofagasta, Antofagasta, Chile; d School of Biological Science, Universiti Sains Malaysia, Persiaran Bukit Jambul, Bayan Lepas, Penang, Malaysia; e Faculty of Science and Natural Resources, Universiti Malaysia Sabah, Kota Kinabalu, Sabah, Malaysia; f Department of Microbiology, Institute of Biology, Bucharest, Romania; University of Delaware

## Abstract

Here, we report the draft genome sequence of *Flavobacterium* sp. strain PL002, isolated from Antarctic *Porphyra* algae. The 4,299,965-bp genome sequence is assembled into 170 contigs, has 32.92% GC content, and 3,734 predicted genes.

## ANNOUNCEMENT

*Flavobacterium* species belonging to the phylum *Bacteroidetes* are Gram-negative, non-endospore-forming, aerobic, oxidase-positive, nonfermenting, yellow-pigmented bacteria inhabiting a wide variety of environments (1). Members of this genus, also described as part of bacterial communities associated with macroalgae from extremely cold environments (Antarctic Ocean), exhibited an interesting capability to hydrolyze phycocolloids (2).

We report here the draft genome sequence of *Flavobacterium* sp. strain PL002, isolated from *Porphyra* (*Rhodophyta*) macroalgae washed up on the shore of Arley Island, Fildes Bay, Antarctica (62°12′30.92″S, 58°56′26.65″W). The isolation was carried out under aseptic conditions using a laminar flow cabinet. The algae were cut into small pieces and washed 3 times with sterile seawater. The cut algae were laid onto a Reasoner’s 2A (R2A; Merck Millipore, Germany) agar medium plate and incubated at 15°C in the dark for 1 week. A single yellow colony that formed a depression on the agar was picked and streaked onto R2A agar medium 3 times to obtain a pure culture. This axenic culture was grown at 15°C on an R2A agar plate for isolation of genomic DNA.

The taxonomic identity of the strain was confirmed by partial sequencing of the 16S rRNA gene. PCR was performed using the 27F (5′-AGAGTTTGATCMTGGCTCAG-3′) and 1492R (5′-TACGGYTACCTTGTTACGACTT-3′) primer pair (3), and the sequencing was conducted using the 341F (5′-CCTACGGGNGGCWGCAG-3′) and 805R (5′-GACTACHVGGGTATCTAATCC-3′) primer pair (4). BLASTn (5) analysis of the 16S rRNA sequence revealed that this strain belonged to the genus *Flavobacterium*. Genomic DNA from *Flavobacterium* sp. strain PL0002 was isolated using a DNeasy blood and tissue kit (Qiagen, Inc., USA) according to the manufacturer’s instructions and followed by PCR amplification of the 16S rRNA gene to confirm its authenticity. The *Flavobacterium* sp. strain PL0002 genomic library was constructed using the Nextera XT DNA sample preparation kit. Whole-genome shotgun sequencing was performed using the 300-cycle MiSeq reagent kit v2, with paired-end 2 × 150-bp reads in the MiSeq sequencing system (Illumina, Inc., San Diego, CA). Sequence reads were filtered using SolexaQA v3.1.3 (6). *De novo* assembly was performed with Velvet v1.2.10 (7). Default parameters were used for all software. Genome sequencing generated 4,464,408 reads with an average length of 151 nucleotide bases (674,125,608 bp in total). *De novo* assembly produced 69 large contigs (>10,000 bp) and 101 smaller contigs (<10,000 bp) with a 125.0× mean coverage and an *N*_50_ contig size of 152,273 bp, *N*_75_ contig size of 88,220 bp, and largest contig size of 234,944 bp. The PL002 genome sequence had an estimated completion of 96.91% (8). Gene prediction and annotation were performed with the Rapid Annotations using Subsystems Technology (RAST) v2.0 server. The resulting PL002 genome sequence showed a GC content of 32.92%, with 3,734 predicted genes, 4 rRNAs, 51 tRNAs, 1 transfer-messenger RNA (tmRNA), and a mean gene length of 978.78 bp.

Multilocus phylogenetic analysis based on maximum likelihood analysis of the concatenated sequences of the genes *atpA*, *dnaK*, *fumC*, *gyrB*, *murG*, *trpB*, and *tuf*, a reliable method for species and strain delineation for *Flavobacterium* taxonomy (9, 10), showed that PL002 had 94.21% sequence identity with Flavobacterium frigidarium strain DSM 17623 (GenBank accession number KE384394.1), 79.31% genome average nucleotide identity (ANIb; based on BLAST), and a DNA-DNA hybridization (DDH) value of 22.9% (11).

### Data availability.

The annotated genome sequence was deposited in DDBJ/ENA/GenBank under the accession number MQTN00000000.1. This project has been submitted under BioProject accession number PRJNA344835 and BioSample accession number SAMN05712601. The raw sequencing data were deposited in the SRA under accession number SRR13311456.
